# Robustness of biomarker determination in breast cancer by RT-qPCR: impact of tumor cell content, DCIS and non-neoplastic breast tissue

**DOI:** 10.1186/s13000-018-0760-6

**Published:** 2018-10-20

**Authors:** Kerstin Hartmann, Kornelia Schlombs, Mark Laible, Claudia Gürtler, Marcus Schmidt, Ugur Sahin, Hans-Anton Lehr

**Affiliations:** 1 BioNTech Diagnostics GmbH, An der Goldgrube 12, 55131 Mainz, Germany; 2 Department of Obstetrics and Gynecology, Johannes Gutenberg University, Langenbeckstraße 1, 55131 Mainz, Germany; 3 BioNTech AG, An der Goldgrube 12, 55131 Mainz, Germany; 4 Institute of Pathology, Medizin Campus Bodensee, Röntgenstraße 2, 88048 Friedrichshafen, Germany

**Keywords:** Breast cancer, MammaTyper, RT-qPCR, DCIS, Tumor cell content, Non-neoplastic tissue, Tissue heterogeneity

## Abstract

**Background:**

Tissue heterogeneity in formalin-fixed paraffin-embedded (FFPE) breast cancer specimens may affect the accuracy of reverse transcription quantitative real-time PCR (RT-qPCR). Herein, we tested the impact of tissue heterogeneity of breast cancer specimen on the RT-qPCR-based gene expression assay MammaTyper®.

**Methods:**

MammaTyper® quantifies the mRNA expression of the four biomarkers *ERBB2*, *ESR1*, *PGR*, and *MKI67.* Based on pre-defined cut-off values, this molecular in vitro diagnostic assay permits binary marker classification and determination of breast cancer subtypes as defined by St Gallen 2013. In this study, we compared data from whole FFPE sections with data obtained in paired RNA samples after enrichment for invasive carcinoma via macro- or laser-capture micro-dissection.

**Results:**

Compared to whole sections, removal of surrounding adipose tissue by macrodissection generated mean absolute 40-ddCq differences of 0.28–0.32 cycles for all four markers, with ≥90% concordant binary classifications. The mean raw marker Cq values in the adipose tissue were delayed by 6 to 7 cycles compared with the tumor-enriched sections, adding a trivial linear fold change of 1.0078 to 1.0156. Comparison of specimens enriched for invasive tumor with whole sections with as few as 20% tumor cell content resulted in mean absolute differences that remained on average below 0.59 Cq. The mean absolute difference between whole sections containing up to 60% ductal carcinoma in situ (DCIS) and specimens after dissection of DCIS was only 0.16–0.25 cycles, although there was a tendency for higher gene expression in DCIS. Observed variations were related to small size of samples and proximity of values to the limit of detection.

**Conclusion:**

Expression of *ESR1, PGR, ERBB2* and *MKI67* by MammaTyper® is robust in clinical FFPE samples. Assay performance was unaffected by adipose tissue and was stable in samples with as few as 20% tumor cell content and up to 60% DCIS.

**Electronic supplementary material:**

The online version of this article (10.1186/s13000-018-0760-6) contains supplementary material, which is available to authorized users.

## Background

Heterogeneity is an intrinsic property of formalin-fixed paraffin-embedded (FFPE) tumor material from core needle biopsies or resection specimens of breast carcinomas. On hematoxylin and eosin (H&E) stained histological slides, invasive tumor cells are seen in close proximity to other neoplastic or non-neoplastic microanatomical structures such as in situ carcinoma, atypical ductal hyperplasia, non-neoplastic ductulo-lobular structures, and stromal cells, including adipocytes, blood vessels, and other cells of the tumor microenvironment. These morphologically distinct cell types have unique biological and molecular fingerprints [1–4].

During the diagnostic work-up of breast carcinomas, immunohistochemistry (IHC) is the standard method for assessing the expression of estrogen- (ER) and progesterone-receptors (PR), human epidermal growth factor receptor 2 (HER2) as well as of Ki-67 as a marker of tumor cell proliferation. Biomarker studies are routinely performed in order to classify breast carcinomas into prognostic and therapeutic categories [5]. The fact that tissue morphology is preserved on IHC-stained slides makes it possible to assess biomarker expression specifically in the invasive tumor compartment, regardless of heterogeneity. However, IHC requires interpretation of the chromogen signal and semi-quantitative scoring of intensity or proportion of staining, procedures that are both subject to intra- and inter-observer variability and will hence result in discordance rates [6–9].

Quantification of gene expression by reverse transcription-quantitative real-time PCR (RT-qPCR) precludes such subjective interpretation. However, contrary to IHC, RT-qPCR uses RNA extracted from FFPE sections, containing both invasive tumor as well as non-tumorous cells of the tumor microenvironment. Therefore, gene expression data may thus be affected by the presence of heterogeneous cell types whose expression patterns can differ substantially from the invasive tumor [3, 4, 10, 11]. With the advent of molecular subtyping of breast cancer and the clinical endorsement of RNA-based genomic risk scores tissue heterogeneity has to be considered a potential confounder and is usually addressed by assay-specific requirements for “minimum tumor content” [12]. Macrodissection or the more time-consuming microdissection is usually applied to increase tumor cell content (TCC) in the diagnostic setting.

The MammaTyper® is an RT-qPCR-based, CE-marked molecular in vitro diagnostic assay used for categorizing tumor resection specimens and pre-operative core needle biopsies of breast carcinomas into five subtypes (*luminal A-like*, *luminal B-like* (HER2-positive), *luminal B-like* (HER2-negative), *HER2-positive* (non-luminal) and *triple negative* (ductal)) as defined by the 2013 St Gallen consensus [13]. The assay quantifies the mRNA expression of four genes *ERBB2* (HER2), *ESR1* (ER), *PGR* (PR) and *MKI67* (marker of proliferation Ki-67) relative to the mean expression of two reference genes and generates a dichotomous result (positive or negative) based on predefined cut-off values [14].

MammaTyper® may evolve as a valid alternative to IHC. This is supported by the substantial correlation that exists between protein and mRNA expression in general [15] and for breast cancer biomarkers in particular [16–18] and by the desire to increase the reproducibility of biomarker testing, in particular for the assessment of proliferative activity (i.e.Ki-67) [19]. MammaTyper® has shown excellent analytical performance, promising clinical validity both in the adjuvant and neoadjuvant setting, with concordance to IHC documented in more than 800 clinical samples [20–22].

During the assay’s technical validation, we have previously studied the robustness of the gene expression assay in the face of tissue heterogeneity [14]. In the present work, we aimed to further examine the impact of tissue heterogeneity on MammaTyper® gene expression by investigating “contamination” at both ends of the histological spectrum. It is commonly assumed that non-neoplastic RNA may solely “dilute” the RT-qPCR signal, whereas in situ carcinoma (i.e.DCIS) may affect results in more complex ways due to its unique transcriptional profiles that differ from that of the invasive tumor [4, 23]. We therefore designed 3 independent experiments to assess the impact of non-neoplastic surrounding tissue on gene expression, in particular adipose tissue and DCIS as well as variations in TCC.

## Methods

### Sample selection and tissue handling

The study consisted of 49 FFPE resection specimens of invasive breast carcinoma. Thirteen cases were from the Institute of Molecular Gynecological Oncology, Mainz, Germany. The use of archived samples was approved by the ethics committee of the Landesärztekammer Rheinland-Pfalz (837.139.05 (4797)). Thirty one cases were obtained from PATH Biobank (Patientsʼ Tumour Bank of Hope), Munich, Germany [24]. Patients provided individual, written informed consent for the storage of samples and data, follow-up contact, and further use of samples and data for research purposes. The processes of PATH Biobank have been approved by the ethics committee of the medical faculty of the University of Bonn (255/06). Five additional cases were purchased from commercial vendors (Asterand Biosciences, Detroit, USA MI; Proteogenex, Culver City, USA CA).

**Fig. 1:**
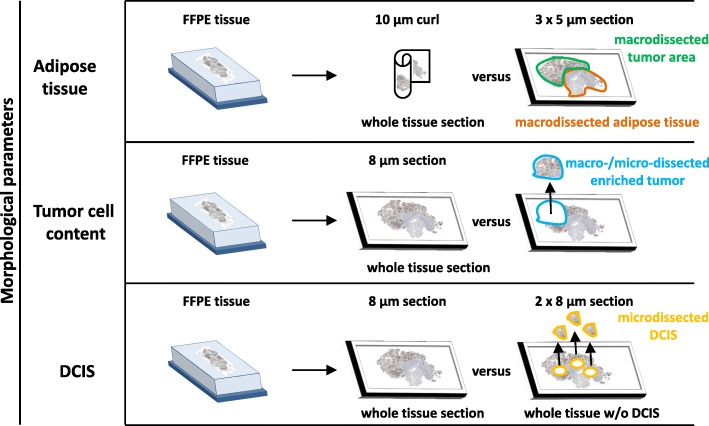
Schematic overview of sample processing and morphological parameters of investigated tissue

Histological review was performed on H&E slides by an experienced pathologist who identified and evaluated the percentage of the various tissue components (non-neoplastic tissue, invasive tumor and DCIS). The effect of adipose tissue, tumor cell content (TCC) and DCIS on the results of MammaTyper® for each individual breast cancer marker was investigated in different experiments (Fig. 1). TCC was defined as the planimetric ratio of areas covered by invasive carcinoma in relation to the area covered by DCIS and by non-neoplastic tissue (including connective and necrotic tissue). Because of its paucicellular nature, scar and adipose tissue were not considered as non-neoplastic tissue. The size of the tumor area (mm^2^) was calculated using the ZEN2 (blue edition) software from Carl Zeiss Microscopy GmbH 2011.

To study the effect of adipose tissue on gene expression, we selected 10 FFPE samples with surrounding adipose tissue which accounted for at least 50% of the whole section. To exclude effects of other tissue components such as non-neoplastic tissue, only samples with 80 to 100% tumor cell content were used. Furthermore, in order to minimize effects of DCIS on assay results, only samples with less than 10% DCIS content were selected for these experiments (8 samples: 0% DCIS; 2 samples: ≤10% DCIS). In addition to a 10 μm curl representing the whole section, three consecutive 5 μm sections were mounted on glass slides and the invasive tumor area of each slide as well as the adipose tissue were macrodissected and transferred into separate RNase-free tubes for subsequent RNA isolation (Fig. 1, upper panel). Relative expression of the four genes *ERBB2*, *ESR1*, *PGR* and *MKI67* in the whole sections was compared with relative gene expression in the dissected invasive tumor and the adipose tissue.

To study the impact of TCC, two 8 μm sections were cut from 15 clinical breast cancer cases with TCC ranging from 20 to 39% (*n* = 7), 40–59% (*n* = 5) and 60–79% (*n* = 3). Like in the experiments on adipose tissue contamination, effects of DCIS on assay results were minimized by selecting only samples with less than 10% DCIS content (9 samples: 0% DCIS; 6 samples: ≤10% DCIS). Sections were mounted on polyethylene naphthalate (PEN) membrane slides, stained with Cresyl violet and enriched up to almost 100% by laser microdissection with a Leica LMD DFC 7000 T (Leica Microsystems) or macrodissection (Fig. 1, middle panel). Sections were then transferred into RNase-free test tubes for subsequent RNA isolation and gene expression studies.

The effect of DCIS on the MammaTyper® results was assessed on 24 FFPE breast cancer samples with DCIS-covered areas ranging from 10 to 60%. The DCIS content was morphologically distinguished from invasive carcinoma via the preservation of the myoepithelial-cell layer, visible by standard H&E staining [25]. From each FFPE sample, three 8 μm sections were prepared for laser microdissection. Circled areas of DCIS from two slides were quantitatively microdissected and combined into an RNase-free tube for RNA isolation. Tissue sections without the microdissected DCIS were transferred in duplicates into RNase-free tubes (Fig. 1, lower panel). Relative expression of the four genes *ERBB2*, *ESR1*, *PGR* and *MKI67* were compared between whole sections, whole sections lacking DCIS and microdissected DCIS. Moreover, 3 μm sctions of these same breast cancer samples were immunostained with an anti-Her2/neu antibody (Clone EP3 Epitomics (Quartett, Berlin, Germany, using DAB as detection medium) in order to assess the Her2 status in invasive tumor and DCIS.

#### RNA isolation and mRNA quantification via RT-qPCR

Extraction of total RNA from FFPE samples was performed using a CE-marked paramagnetic bead-based method (RNXtract®, BioNTech Diagnostics, Mainz, Germany) according to the manufacturers’ instructions. RNA was eluted in 100 μl, 60 μl or 50 μl depending on the amount of input material. The median gene expression levels of both reference genes measured within the MammaTyper® test were used as a quality measure for determining the adequacy of the amount of RNA present in the sample.

The expression levels of *ERBB2*, *ESR1*, *PGR* and *MKI67* were determined by reverse transcription-quantitative real-time PCR (RT-qPCR) using the CE-marked MammaTyper® IVD kit (BioNTech Diagnostics, Mainz, Germany) on the LightCycler® 480 II qPCR platform (Roche Diagnostics) according to the manufacturer’s instructions (160301–90020-EN Rev. 3.1). Calculations were carried out as described previously [14]. MammaTyper® results are expressed as 40-ddCq values for each marker which represent the gene expression level in the sample relative to the amount of RNA starting material as determined by the reference genes beta-2-microglobulin (*B2M)* and calmodulin 2 (*CALM2)*. In addition, each individual marker was scored positive or negative according to clinically validated marker specific cutoffs [20–22]. The following cut-offs were used: *ERBB2* 41.10, *ESR1* 38.00, *PGR* 35.50, *MKI67* 35.90.

## Results

### Effect of adipose tissue on gene expression studies

**Fig. 2:**
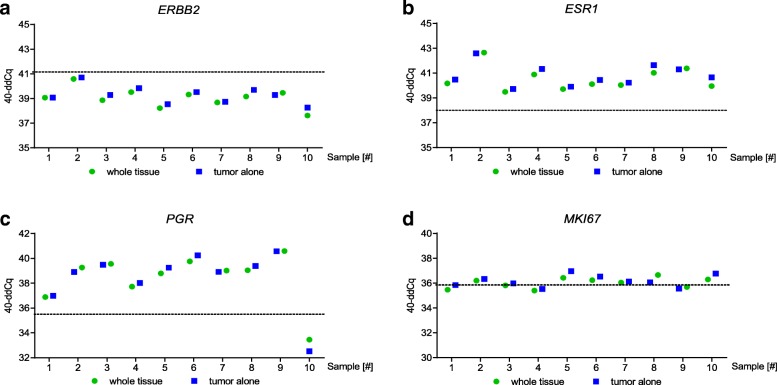
Effect of adipose tissue on relative gene expression. Shown in the graph are gene expression data of *n* = 10 breast cancer specimen for *ERBB2*(**a**), *ESR1* (**b**), *PGR* (**c**) and *MKI67* (**d**) in whole sections (over 50% adipose tissue content, green circles) versus tumor-enriched sections (blue squares). Dotted lines represent the respective cut-off for the four marker genes (*ERBB2*: 41.10; *ESR1*: 38.00; *PGR*: 35.50; *MKI67*: 35.90)

Due to the high ratio of cytoplasm over nuclei in adipose tissue, and therefore low cellularity, the differences of 40-ddCq values across the tested pairs of whole sections and samples obtained after removing the surrounding adipose tissue were low. The mean absolute difference (and min/max observed value) was 0.28 cycles for *ERBB2* (0.00–0.64), 0.31 for *ESR1* (0.05–0.70), 0.32 for *PGR* (0.02–0.94), and 0.29 for *MKI67* (0.09–0.59) (Fig. 2, Additional file 1). On average, the difference was even smaller than the typical intra-run variation of 0.5 cycles observed in qPCR experiments (corresponding to a standard deviation (SD) of 0.35 cycles) [26]. The expected SD of Cq values is even higher (0.4 cycles) in the region of the limit of detection (LOD) of a qPCR [27]. The concordance of the binary categories was 100% for *ERBB2*, *ESR1* and *PGR* and 90% for *MKI67* caused by one single case where the initial value was very close to the cut-off (Additional file 1, sample 3). The highest difference (0.94 cycles) was detected in a single case with *PGR* gene expression close to the limit of detection in the dissected (tumor-enriched) sample (Additional file 1, sample 10).

Four out of 10 macrodissected adipose-enriched tissue samples had invalid MammaTyper® results, because the RNA yield was poor, as evidenced by the very low expression levels of the reference genes. In the remaining 6 samples, the results were valid but borderline, with reference Cq values close to the limit of detection. The signal of the reference genes in the pooled adipose tissue was detected on average 4 cycles later than the signal from the invasive tumor area. Regarding the marker genes, the mean raw Cq values in the adipose tissue pools were delayed by 6 to 7 cycles compared with the tumor-enriched tissue, corresponding to a 2^− 6^ to 2^− 7^ change adding a linear fold change of 1.0156 or 1.0078, respectively. This results in a negligible Cq change of − 0.011 to − 0.022 (Additional file 2).

### Effect of tumor cell content on gene expression studies

**Fig. 3:**
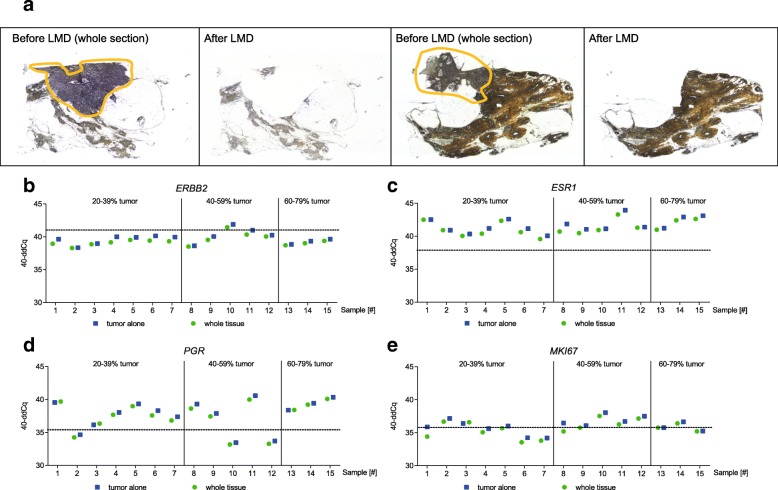
Effect of normal tissue and TCC on relative gene expression. **a** Representative images of sample number 9 (left) and sample number 3 (right) before and after microdissection of the respective tumor area (encircled in orange). **b**-**e** Shown in the graph are gene expression data of *n* = 15 breast cancer specimen for *ERBB2*(**b**), *ESR1* (**c**), *PGR* (**d**) and *MKI67* (**e**) in whole sections (green circles) versus tumor-enriched sections (blue squares). Dotted lines represent the respective cut-off for the four marker genes (*ERBB2*: 41.10; *ESR1*: 38.00; *PGR*: 35.50; *MKI67*: 35.90)

The mean absolute difference in relative gene expression between samples before and after enrichment for invasive tumor was low (< 0.59 Cq) (Table 1 and Fig. 3). In 7 out of 60 measurements (11.7%) the single marker results showed an absolute difference which was higher than 0.70 Cq, the typical intra-run variation (2x SD of 0.35 cycles) observed in experiments with qPCR [26]. These deviations were particularly observed in 4 very small samples with a tumor area less than or equal to 25 mm^2^ (Additional file 3) and showed raw Cq values close to the LOD for some markers.

**Table 1:** Differences in MammaTyper® relative gene expression between pairs of whole tissue and tumor-enriched specimens

Tumor cell content	Mean (min, max) absolute difference of 40-ddCq of paired measurements			
	*ERBB2*	*ESR1*	*PGR*	*MKI67*
20–39%	0.49 (0.04 to 0.86)	0.34 (0.01 to 0.80)	0.39 (0.15 to 0.72)	0.58 (0.17 to 1.46)
40–59%	0.38 (0.11 to 0.65)	0.53 (0.08 to 1.15)	0.49 (0.28 to 0.68)	0.58 (0.33 to 1.28)
60–79%	0.24 (0.15 to 0.30)	0.40 (0.22 to 0.50)	0.16 (0.04 to 0.23)	0.09 (0.01 to 0.23)

The binary categories were discordant in 3 *MKI67* cases, having 40-ddCq values adjacent to the cut-off (Additional file 3, sample 5, 8 and 9). As a consequence, 3 *Luminal A-like* samples, one with 20–39% TCC and 2 with a TCC of 40–59% were re-classified as *Luminal B-like* (*HER2-negative*) after tumor enrichment. The pairs of dissected and non-dissected samples for the other markers showed a concordance rate of 100%.

### Effect of variable extent of DCIS on gene expression studies

Sample characteristics used for this gene expression study are summarized in Table 2.

**Table 2:** Characteristics of tissue samples used for the analysis of DCIS on relative gene expression

Sample #	Tumor cell content [%]	DCIS content [%]	HER2 status (IHC)	
			invasive tumor	DCIS
1	40–59	**10–19**	3+	3+
2	60–79		3+	3+
3	80–100		3+	3+
4	80–100		n.a.	n.a.
5	20–39		2+	2+
6	60–79		**2+**	**3+**
7	60–79	**20–29**	0	0
8	60–79		0	0
9	40–59		**0**	**1+**
10	40–59		0	0
11	20–39		1+	1+
12	40–59		1+	1+
13	40–59		**1+**	**2+**
14	40–59		0	0
15	60–79		n.a.	n.a.
16	60–79		1+	1+
17	20–39	**30–39**	**0**	**1+**
18	20–39		**2+**	**3+**
19	20–39		0	0
20	20–39		**1+**	**2+**
21	40–59	**40–49**	1+	1+
22	20–39	**50–60**	**1+**	**2+**
23	20–39		**1+**	**2+**
24	40–59		1+	1+

Different scores of HER2 protein expression in DCIS and corresponding invasive tumor are shown in bold

**Fig. 4:**
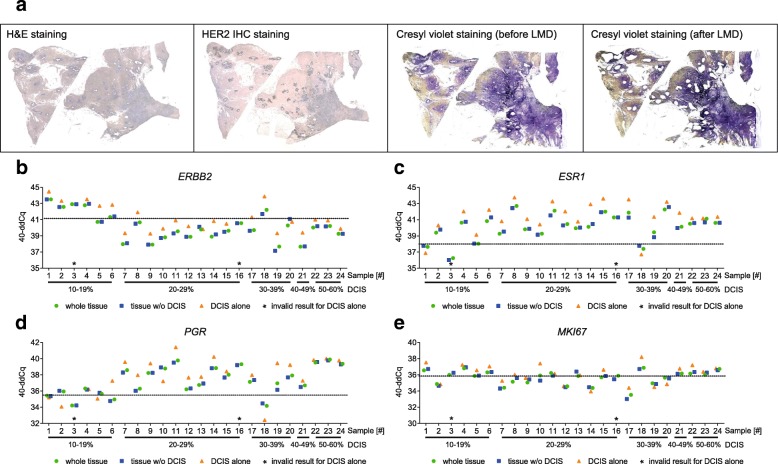
Effect of DCIS on relative gene expression. **a** Representative images (sample 17) of H&E stained, HER2 immunostained and cresyl violet stained sections before and after microdissection of DCIS-only areas. **b**-**e** Shown in the graph are gene expression data for *n* = 24 breast cancer specimen for *ERBB2* (**b**), *ESR1* (**c**), *PGR* (**d**) and *MKI67* (**e**) of paired whole tissue sections (green circles), whole tissue sections without DCIS (blue squares) and DCIS-enriched (orange triangles) microdissected tissue. Dotted lines represent the respective cut-off for the four marker genes. (*ERBB2*: 41.10; *ESR1*: 38.00; *PGR*: 35.50; *MKI67*: 35.90)

40-ddCq differences across the tested pairs of whole tissue and microdissected tumor samples without DCIS were low for all four genes (Fig. 4). The mean absolute difference was 0.16 Cq for *ERBB2* (0.00 to 0.79 Cq), 0.25 for *ESR1* (0.00 to 0.61 Cq), 0.18 for *PGR* (0.01 to 0.80) and 0.24 for *MKI67* (0.03 to 0.63 Cq) (Additional file 4). When DCIS-only samples were separately analyzed, expression profiles tended to be higher (compared to invasive tumor), exceeding the generally accepted intra-run variation of 0.70 cycles in 18/22 cases for *ERBB2* (81.8%), in 19/22 cases for *ESR1* (86.4%), in 14/22 cases for *PGR* (63.6%) and in 11/22 cases for *MKI67* (50.0%). In four cases (Table 2, samples 6, 9, 17 and 18), HER2 protein expression by immunohistochemistry showed a similar trend towards higher scores in DCIS as the one observed for gene expression studies (Fig. 4b and Table 2). This trend can be especially seen in cases that turn from 2+ (invasive tumor) to 3+ (DCIS) and from 0 (invasive tumor) to 1+ (DCIS). In 2 of 24 DCIS-only samples, gene expression studies were invalid due to insufficient RNA yield.

## Discussion

The gene expression profiles of prognostic and predictive biomarkers in breast cancer likely differ between invasive carcinoma, non-invasive carcinoma (e.g. DCIS), non-neoplastic ductulo-lobular units, and adjacent or intervening stroma. Whole FFPE sections of breast cancinomas contain variable amounts of such tissue “contaminants”. In this study, we investigated the robustness of MammaTyper®, an RT-qPCR-based gene-expression assay for *ERBB2*, *ESR1*, *PGR* and *MKI67* against heterogeneity due to various tissue types. We first focused on the surrounding adipose tissue, which can easily be removed by macrodissection. By measuring the expression of reference genes we showed that the total RNA obtained from adipose tissue was on average more than 10-fold lower (3.5 Cqs) than the RNA isolated from equal volumes of invasive tumor tissue, frequently falling below the limit of detection. Even when adipose tissue occupied more than 50% of the slide area, deviations of gene expression between whole sections and sections lacking the surrounding tissue fell in the range of intra-run variance.

The observation that adipose tissue contains far less RNA than similarly sized tumor tissue is explained by the fact that adipocytes have very small nucleocytoplasmic ratios (small nuclei, voluminous cytoplasm) [28]. By contrast, cancer cells tend to exhibit a particularly high nucleocytoplasmic ratio due to their increased DNA content and reduced specialized cytoplasmic organelles. Hence, invasive tumor areas have significantly more nuclei per mm^2^ than surrounding non-malignant breast tissue and thus considerably larger amounts of nucleic acids. Our experiments indicate that this phenomenon is so pronounced as to trivialize the impact of RNA derived from adipose tissue on the total RNA yield of the non-dissected whole sections. The fact that RNA yields from normal breast tissue which consists largely of adipose and paucicellular fibrous tissue are often insufficient is well known and poses a challenge for studies requiring adequate non-neoplastic control material [4, 29].

Due to the negligible influence of adipose tissue on gene expression for *ERBB2*, *ESR1*, *PGR* and *MKI67*, this component was disregarded for the calculations of the TCC. In the respective experiments, we found that changes in the relative gene expression were insignificant down to 20–39% TCC. This is in agreement with data reported by Tramm and coworkers, who showed an excellent agreement between gene expression data for *ESR1, PGR* and *ERBB2* from whole tissue and from macrodissected extracts [30]. Likewise, macrodissection did not affect the prognostic significance of RNA expression of cancer-associated genes in primary breast tumor samples [12]. Moreover, Viale and coworkers showed that discordances between RNA microarray readouts and IHC/FISH for ER, PGR and HER2 in the MINDACT trial could not be explained by intratumoral heterogeneity or the presence of either DCIS or normal tissue [31]. In line with that, other breast cancer assays, like the GeneXpert Breast Cancer STRAT4 assay from Cepheid, that also measures the expression levels of *ERBB2*, *ESR1*, *PGR* and *MKI67*, found that macrodissection of whole tissue sections is not required for accurate assessment of these genes by RT-qPCR [32].

Contrary to the findings from studies looking at the effect of TCC on the expression of individual genes, Elloumi and coworkers found that multi-gene genomic scores were susceptible to contamination of RNA eluates by normal breast tissue [33]. However, specimen volume was not normalized, which may explain why the impact of non-tumor tissue on the expression levels of the genes of interest may have been overestimated in their study. Along the same line of reasoning, first results for the Prosigna® gene signature were unstable if samples contained more than 60–70% surrounding non-tumor tissue [34]. The question whether multigene risk predictors are sensitive or not to variations of TCC is hence related to factors like test design and gene-specific ratios of tumor-vs-normal expression.

Data from studies with complex multigene predictors may not adequately address the impact of TCC on gene expression, as they depend on specificities of different genes and their particular mode of expression in different tissue compartments. For example, the outcome estimations based on *MMP7*, a gene which encodes for an enzyme that degrades extracellular proteins, were discordant before and after macrodissection [2], most likely because RNA expression of *MMP7* is higher in stroma compared to tumor cells [35]. Conversely, ERa, the clinically relevant isoform of the estrogen receptor is confined to epithelial cells of the breast and is not expressed in mammary fibroblasts [36]. Thus, the impact of TCC on RNA relative quantification is gene-specific and as far as MammaTyper® genes *ERBB2, ESR1, PGR* and *MKI67* are concerned the bias introduced by the inclusion of tissue surrounding the invasive tumor appears to be non-critical.

The present study underscores the fact that caution must be applied when analyzing samples that are critically small in size and hence yield only low amounts of RNA. Depending on how close the respective value lies to the limit of detection, gene expression may be affected by tumor cell enrichment.

It is well documented that gene expression patterns and molecular breast cancer subtypes may vary considerably between invasive tumor and DCIS [4, 23]. In keeping with diagnostic anatomo-pathological experience, the relative expression of *ERBB2*, *ESR1*, *PGR* and *MKI67* in our study was often higher in DCIS samples than in samples enriched for invasive carcinomas. Why DCIS is nevertheless only a weak contaminant may be explained by the reduced cellularity of DCIS vs. invasive carcinomas due to cribriform and clinging architecture as well as central necrosis. Others have previously shown that the mean RNA recovery from DCIS was substantially lower than that of invasive tumor of similar volume [1–4]. Thus, it appears that DCIS does not bias gene expression of *ERBB2*, *ESR1*, *PGR* and *MKI67* because the contributory gene expression of DCIS is diluted.

Compared to previous work exploring the significance of TCC on the stability of gene expression assays, our present study has the advantage of addressing biological diversity of whole sections which underlies the apparent histological heterogeneity. Eventually, whether surrounding adipose tissue, normal tissue adjacent to tumor or admixed DCIS, the RT-qPCR signal is dominated by the invasive tumor component, allowing for consistent calculations in whole sections with up to 60% DCIS and in a TCC range of 20–100%. This TCC range remains sufficient even if the tissue area excluding adipose tissue occupies less than 50% of the whole section, indicating that macrodissection of surrounding adipose tissue is not required. Moreover, by using archived material from actual patient cases, our data are meaningful for use in a real-life routine pathology diagnostic setting.

## Conclusion

Our data indicate that MammaTyper® is capable of tolerating low-purity input material with a minimum TCC of 20%. Based on these thresholds, the assay can be used for the robust quantification of *ERBB2*, *ESR1*, *PGR* and *MKI67* on whole sections of FFPE samples during routine histopathological work-up of breast carcinoma.

## Additional files

Additional file 1: Mammatyper® relative gene expression and Δ40-ddCq values are shown for the effect of adipose tissue. (XLSX 11 kb)

Additional file 2: Mammatyper® Median Cq values are shown for the effect of adipose tissue. (XLSX 16 kb)

Additional file 3: Mammatyper® relative gene expression and Δ40-ddCq values are shown for the effect of TCC. (XLSX 13 kb)

Additional file 4: Mammatyper® relative gene expression and Δ40-ddCq values are shown for the effect of DCIS. Tumor cell content, DCIS content, HER2 immunohistochemical score of the invasive tumor and the DCIS, as well as relative expression of the mRNA markers *ERBB2*, *ESR1*, *PGR* and *MKI67* and absolute Δ40-ddCq values are shown. (XLSX 16 kb)

## Abbreviations

B2M: beta-2-microglobulin
CALM2: calmodulin 2
Cq: quantification cycle
DCIS: ductal carcinoma in situ
ER: estrogen receptor
ERBB2: human epidermal growth factor receptor 2
ESR1: estrogen receptor 1
FFPE: formalin-fixed paraffin-embedded
H&E: hematoxylin and eosin
HER2: human epidermal growth factor receptor 2
IHC: immunhistochemistry
IVD: in vitro diagnostic
LMD: laser microdissection
MKI67: marker of proliferation Ki-67
mRNA: messenger RNA
PEN: polyethylene naphthalate
PGR: progesterone receptor
PR: progesterone receptor
RT-qPCR: reverse transcription-quantitative real time PCR
TCC: tumor cell content
